# Uterine Arteriovenous Malformation: Approach and Treatment in a Nulligravidous Virgin Woman

**DOI:** 10.7759/cureus.77287

**Published:** 2025-01-11

**Authors:** Fatima Ba Khamis, Bedayah Amro

**Affiliations:** 1 Obstetrics and Gynecology, Dubai Health, Dubai, ARE; 2 Gynecology, Latifa Hospital, Dubai, ARE

**Keywords:** interventional radiology-guided embolization, uavm, uterine arteriovenous malformations, uterine artery embolization (uae), uterine av malformation

## Abstract

Uterine arteriovenous malformation is a rare condition characterized by abnormal connections between the arteries and veins in the uterus. This condition, though uncommon, can have severe implications for women’s reproductive health and requires prompt diagnosis and appropriate treatment. This case report presents a nulliparous 20-year-old virgin patient with congenital arteriovenous malformation who wanted to preserve her fertility and was refusing all vaginal approaches for diagnosis and treatment. She was admitted to our facility and had two attacks of profuse vaginal bleeding, in which she lost 3 L of blood. She was diagnosed by a pelvic CT scan and treated by bilateral uterine artery coil embolization. To our knowledge, this is the first reported case of a patient presenting with profuse vaginal bleeding who was both nulliparous and a virgin.

## Introduction

Uterine arteriovenous malformation (UAVM) directly connects the arterial system to the venous system without the contribution of capillary vessels. This vascular abnormality can occur in other organs, such as the brain. However, its presence in the uterus is rare, with just over a hundred reported in the literature. The first case was reported in 1926 by Dubreuil and Loubat [1].

Although the exact cause of UAVM remains unclear, several factors have been identified to be associated with this disease. Most reported cases of UAVM have been in women who had been pregnant before or had undergone recent uterine surgery, such as dilation and curettage, cesarean section, or other gynecological procedures that might lead to trauma and injury to the uterus.

Hormonal fluctuation during pregnancy and menstruation has been linked to the pathogenesis of UAVM. Notably, estrogen-mediated notch signaling in the endothelial cells is believed to play an essential role in developing arteriovenous malformations (AVMs). Therefore, it is theorized that the appearance of UAVM can be linked to estrogen levels during pregnancy [2]. UAVM is also believed to rarely be caused by congenital malformations due to abnormalities in the embryological development of the uterine blood vessels.

Patients with this condition usually present with a variety of symptoms, although some women may not experience any symptoms. Common signs and symptoms include abnormal vaginal bleeding, pelvic pain, or discomfort [3].

Diagnosing UAVM can initially be done using transvaginal ultrasound, which should be re-evaluated by Doppler ultrasound as it differentiates UAVM from other similar conditions, such as gestational trophoblastic disease and retained product of conception, using sound waves to measure blood flow. A Doppler ultrasound can detect abnormal blood vessel connections. MRI is another non-invasive diagnostic method that can provide detailed images of the uterus and help identify UAVM. Angiography, even though an invasive method, remains the gold standard for diagnostic evaluation [4].

UAVM can present challenges in its management, but various treatment options are available. The choice of treatment depends on symptom severity, desire for fertility preservation, and overall health considerations. Treatment options include conservative management in stable patients with no bleeding. The medical approach can be used for patients with no history of heavy vaginal bleeding who can be followed up. These medications include gonadotropin-releasing hormone (GnRH) agonists as they downregulate estrogen secretion, which has been linked to the formation of UAVM leading to increased uterine arterial resistance and decreased uterine blood flow [2]. Alternative options include using a combined oral contraceptive pill (COCP) or administering methylergonovine maleate. Documented cases in the literature have shown partial or complete resolution of the lesions with treatment using GnRH analogs, COCPs, and methylergonovine maleate [4,5]. An added benefit of this medical approach is the potential for fertility preservation, with published reports of successful pregnancies following treatment with GnRH analogs, COCPs, and medroxyprogesterone acetate [5,6]. However, a limitation is that it may only be appropriate as a primary treatment for hemodynamically stable patients.

With the evolution of the interventional radiology field, uterine artery embolization (UAE) has become a frequently used minimally invasive procedure. UAE has a faster recovery period and can usually be performed under local anesthesia, avoiding the risks of general anesthesia compared to surgical approaches. However, it carries a risk of radiation exposure.

Surgical interventions include laparoscopic ligation of the internal iliac artery with non-resorbable clips or surgical ligation of uterine arteries with or without laparoscopic myometrial lesion resection, especially in patients suffering from persistent bleeding post-transcatheter embolization [7]. However, minimally invasive surgeries may be the mainstay of care in some patients with no further re-treatment [8]. In more severe presentations, given that the patient has completed their family, a hysterectomy should be considered.

Regarding fertility-sparing options for UAVM, UAE, a much-performed procedure [9], is described in some studies as the gold-standard approach [10]. However, emerging evidence suggests that hysteroscopic resection of the lesions may offer superior outcomes in preserving fertility and overall pregnancy outcomes compared to UAE [9].

Temporary measures to control bleeding in hemodynamically unstable patients include uterine packing and Foley catheter insertion and administering medications such as 15-methyl-prostaglandin F2α, parenteral estrogen and progestin, methylergonovine, and danazol [4].

This case report presents a nulliparous 20-year-old virgin patient with congenital AVM who wanted to preserve her fertility and was refusing all vaginal approaches for diagnosis and treatment. She was initially diagnosed by a pelvic CT scan and was treated successfully by bilateral uterine artery coil embolization.

## Case presentation

A 20-year-old, thin-built, previously healthy woman of Middle Eastern descent, who had never been sexually active, presented to our emergency department with severe vaginal bleeding associated with passing large clots. She provided a history of similar concerns two months back, for which she went to another hospital facility and received multiple units of blood transfusion. At the facility, she received tranexamic acid, which was complicated by an anaphylactic reaction. Her family history was positive for breast cancer, but there was no history of bleeding disorders.

She brought a report of a pelvic CT scan with contrast. The findings included a posterior uterine wall vascular mass lesion protruding and distending the uterine cavity, multiple mildly dilated tufts of vessels with intense enhancement, and the post-contrast arterial phase associated with early filling of multiple venous collaterals, likely representing the nidus. Bilateral dilated tortuous pelvic venous channels were more evident on the left side, likely draining into the internal iliac veins. The findings were highly suggestive of UAVM. Unfortunately, images of this study were not available.

**Figure 1:**
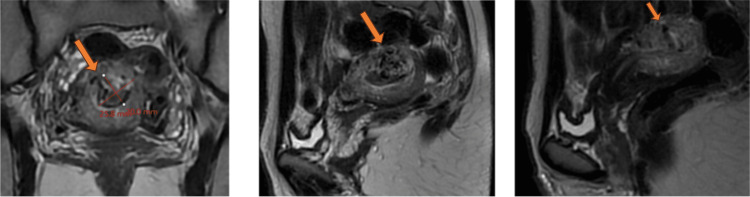
MRI of the pelvis with contrast showing the uterus is retroverted measuring 8.5 × 8 × 4 cm. Intrauterine intracavitary mass-like lesion measures 3 × 2.7 × 2.5 cm, collectively formed of multiple serpentine flow-related signal voids. It extends from the right parametrium through the anterior uterine wall mid-corporal to the endometrial cavity extending further to the left parametrium. A post-contrast examination shows the abnormal complex enhancement of serpentine vessels. Findings are suggestive of uterine arteriovenous malformation.

Upon presentation to our facility, the patient had lost around 1 L of blood, her hemoglobin was 7.5 mg/dL, and she was vitally stable. Hence, two units of blood transfusion were administered, and repeated hemoglobin was 10.1 mg/dL. MRI of the pelvis with which showed findings suggestive of UAVM (Figure 1).

A few hours after admission, the patient suffered another sudden episode of profuse vaginal bleeding with an estimated blood loss of 2 L, for which she needed intensive care. She received three units of blood transfusion along with two units of fresh frozen plasma, 3 mg of Factor VIIa, and 10 units of cryoprecipitates. As bleeding was still ongoing, insertion of a Foley catheter as uterine tamponade was considered; however, the patient ultimately refused the vaginal approach.

**Figure 2:**
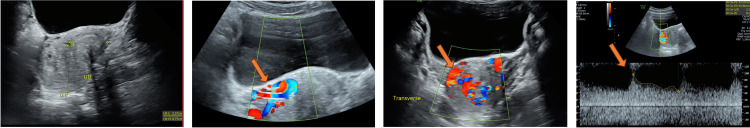
Uterus is normal in size with abnormal myometrium having multiple feeding arteries, a tangle of vessels, and numerous large draining veins favoring a diagnosis of congenital arteriovenous malformation. Color Doppler shows vessels with low resistive index and high peak systolic velocity flow pattern. Both ovaries are normal in size (right ovary: 2.2 × 2.7 cm; left ovary: 1.4 × 1.9 cm) with normal echotexture and no focal lesions. Both adnexa are free from mass, but tubular vascular channels are profound. There is no free fluid in the pelvis.

The bleeding resolved spontaneously within two hours, and her repeated hemoglobin was 12.7 mg/dL. In our facility, a transabdominal ultrasound scan was obtained. The findings were consistent with uterine congenital AVM (Figure 2).

After stabilizing her condition with medications, the patient was counseled regarding different management plans available in our tertiary hospital setting. She expressed a preference for a fertility-sparing option and refused all vaginal approaches, including hysteroscopic resection of the lesions. She was offered UAE after involving the interventional radiology and vascular surgery teams and agreed. The patient underwent selective bilateral UAE. During the procedure bilateral uterine arteries were observed directly shunting into the veins, confirming the diagnosis of an AVM.

**Figure 3:**
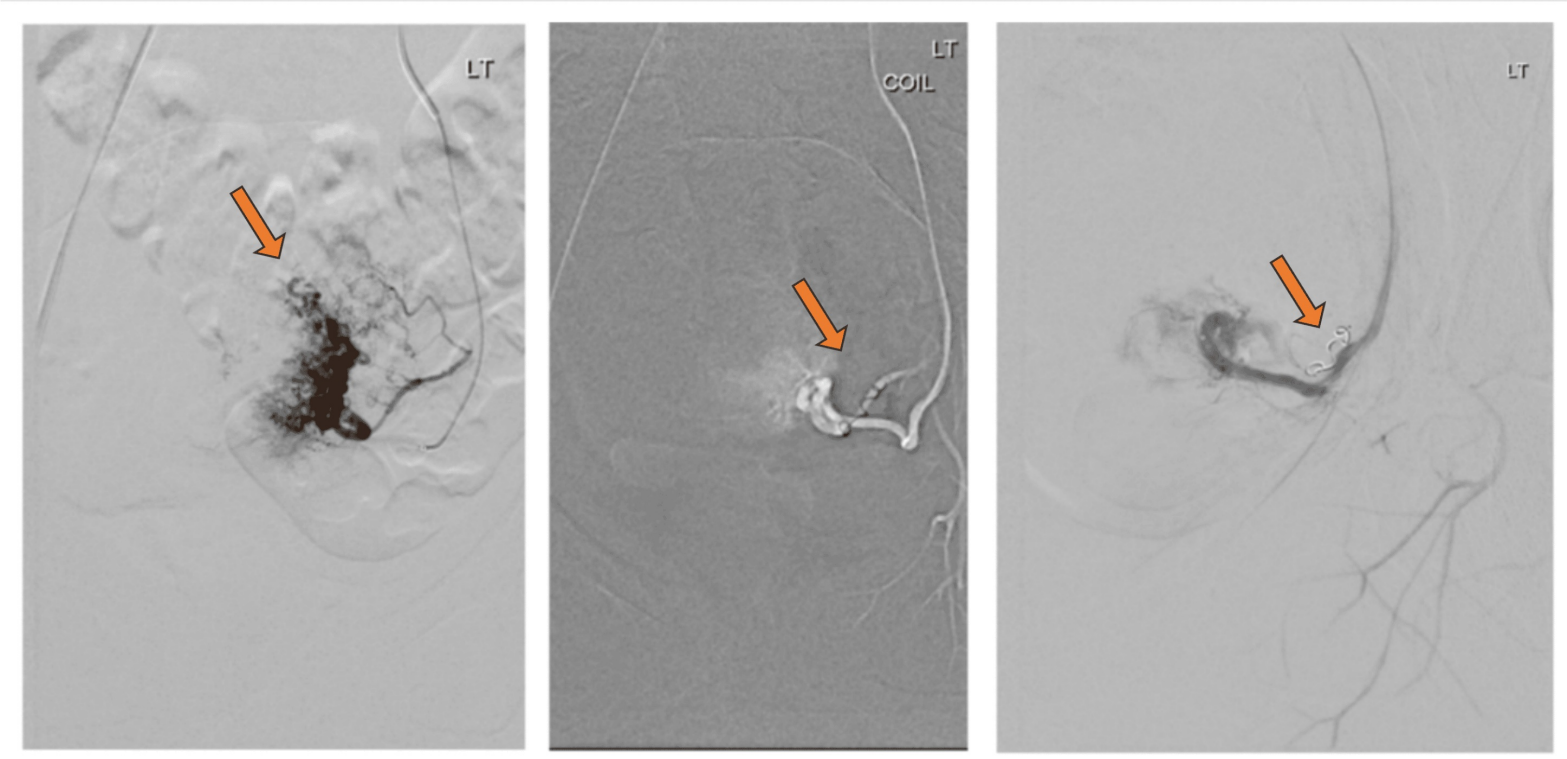
An angiogram of the left uterine artery showing multiple irregular pathological vessels. The coil was inserted at the origin of the anastomotic branch connecting the left ovarian artery to the left uterine artery and embolized using microparticles.

An anastomotic branch connecting the left ovarian artery to the left uterine artery was identified. To reduce blood flow shunting to the left uterine artery and prevent retrograde reflux of the embolization material into the left ovarian artery, which could potentially cause left ovarian necrosis, a coil was placed at the origin of the anastomotic branch. Following this, the microcatheter was advanced until reaching the left uterine artery and microparticles were used to embolize it (Figure 3).

**Figure 4:**
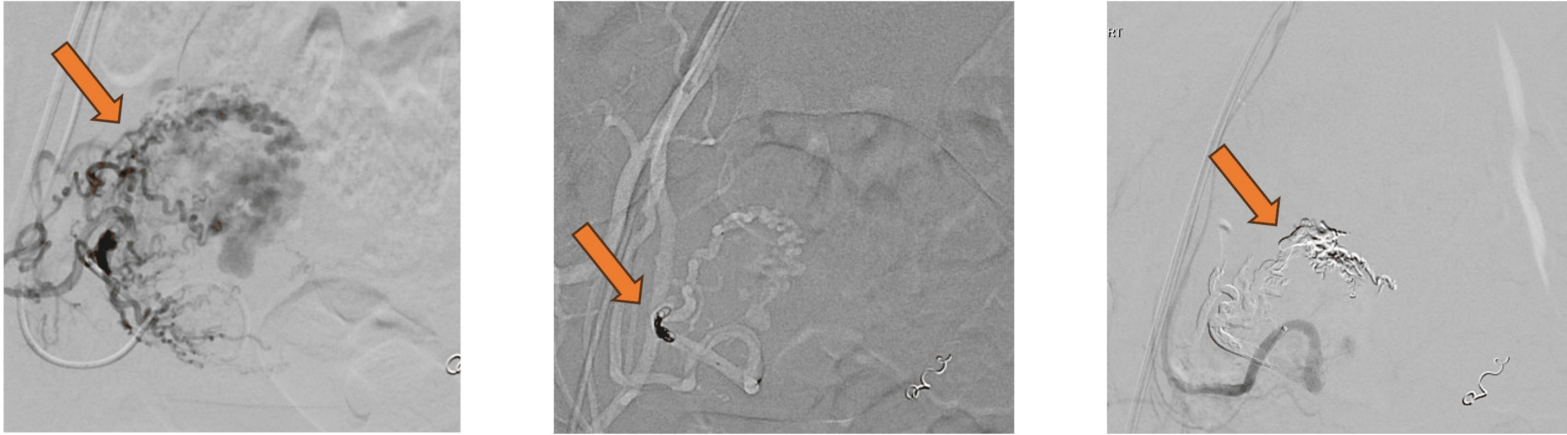
Right uterine artery angiogram showing hypertrophic right uterine artery with multiple pathological vessels with direct shunting into the veins. The coil was inserted in the right uterine artery and then the uterine artery was embolized using Onyx.

**Figure 5:**
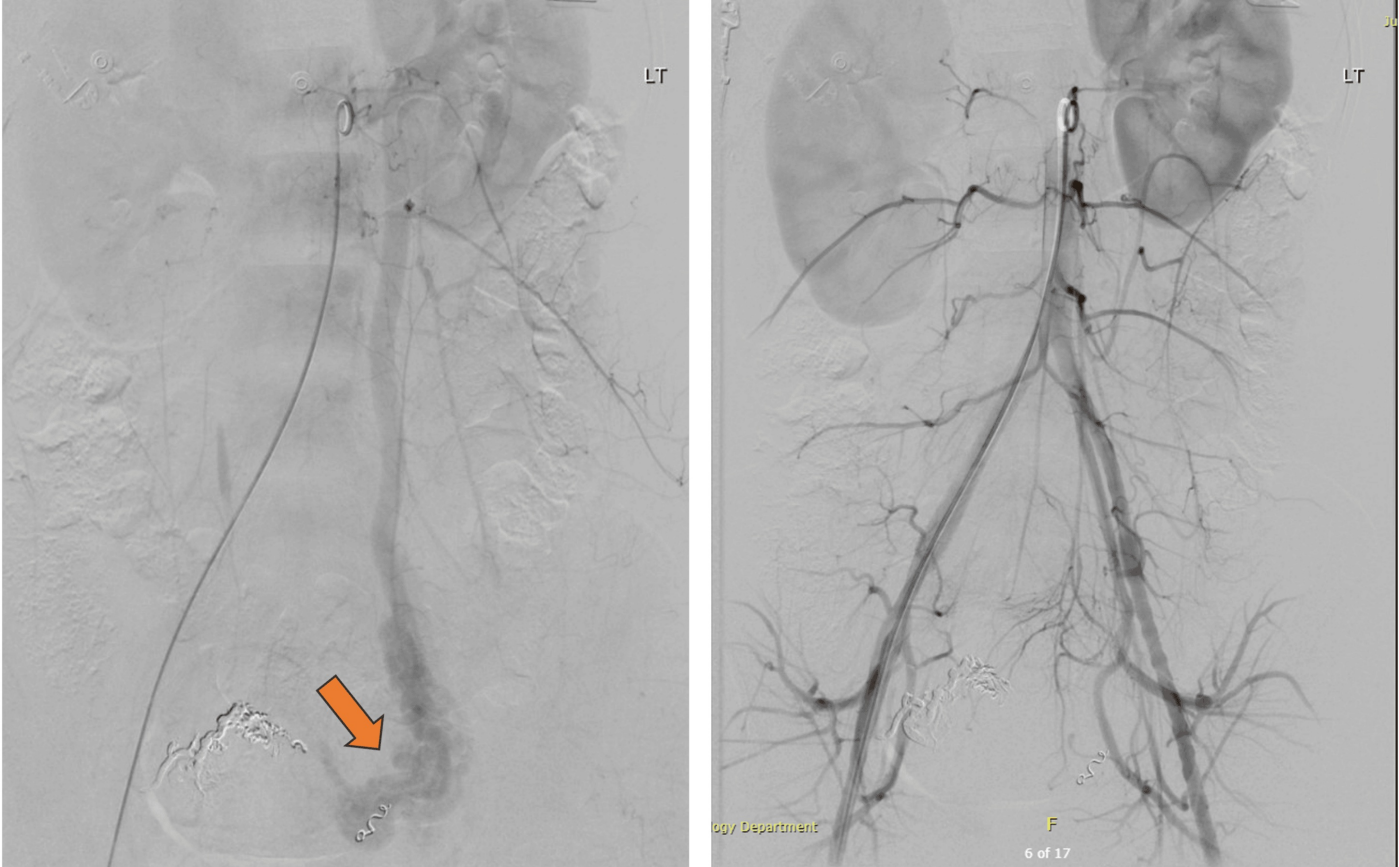
Post embolization angiogram showing complete occlusion of the pathological arteries feeding the UAVM, reverse flow within the left ovarian vein causing varicosity within the pelvis is identified.

On the right side, a coil was inserted at the origin of the right uterine artery to slow the flow within the feeding vessel, followed by using Onyx, a non-adhesive liquid embolic agent (Figure 4). Post-embolization angiogram showed complete occlusion of the pathological arteries feeding the AVM (Figure 5). A pigtail catheter was advanced into the abdominal aorta. An angiogram showed reverse flow within the left ovarian vein causing varicosity within the pelvis in the venous phase (Figure 5). The patient tolerated the procedure well.

Post-embolization angiogram showed complete occlusion of the pathological arteries feeding the AVM (Figure 5). The remained stable with no further episodes of heavy vaginal bleeding, fever, or significant pain and was discharged four days after the procedure.

**Figure 6:**
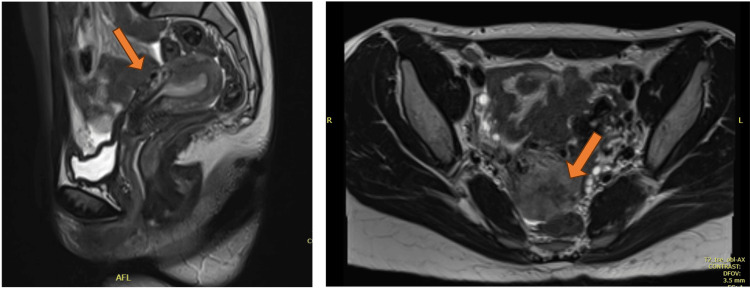
Pelvis MRI with contrast post-uterine artery embolization showing a thrombsed arteriovenous malformation in the right parametrium and left parametrial serpentine vascular channels with contrast enhancement, suggestive of persistent activity.

The patient underwent an MRI of the pelvis with contrast two months after the UAE procedure (Figure 6). The MRI showed right parametrium and anterior uterine wall myometrium AVM with no noticeable contrast enhancement or restricted diffusion, likely thrombosed AVM, and left parametrial serpentine vascular channels with contrast enhancement, suggestive of persistent activity.

She underwent three-month follow-ups in the gynecology clinic and reported having regular periods with heavy flow only on the second day and no further complaints. Her anti-Müllerian hormone level was 4.52 ng/mL and follicle-stimulating hormone was 5.5 mlU/mL, which were within the normal range for pre-menopausal women. She was advised to undergo hysteroscopic resection to control her bleeding; however, she refused.

The patient was called again after one year of the UAE procedure as she was lost to follow-up and reported regular, normal flow periods with no complaints apart from chronic mild low back pain managed with mefenamic acid.

## Discussion

UAVM is a rare but potentially serious condition that can lead to profuse vaginal bleeding. Early diagnosis and appropriate treatment are crucial for managing UAVM and preserving a woman’s reproductive function.

Although many hypotheses have been proposed for the development of UAVM, in general, the etiology of UAVM can be classified as either congenital or acquired types. The acquired types hypothesize hormonal fluctuations during pregnancy or menstruation, particularly estrogen or previous uterine trauma, to be the cause of the condition, as evidenced by most previously reported cases of UAVM in women of the reproductive age group who had been pregnant before or had undergone previous uterine surgery, such as curettage.

Even though extremely rare, embryologically arrested vascular development is also believed to lead to anomalous differentiation in the capillaries and abnormal communication between arteries and veins. Most patients present with bleeding following pregnancy or induced abortion, with remarkably few reports of nulligravida women presenting with the condition.

To our knowledge, this is the first published case of a patient presenting with profuse vaginal bleeding due to UAVM who was both nulliparous and a virgin. We believe that our patient had congenital UAVM, given the absence of known risk factors associated with the acquired type. Congenital AVM is a rare condition, and it is even rarer for it to present as bleeding in a nulligravida patient. Hence, despite the rarity, the condition should be added to the differential diagnosis of severe vaginal bleeding.

As the patient had not had a sexual encounter before and considering her cultural background, it likely contributed to the patient’s strong desire to preserve her hymen’s integrity. Despite experiencing multiple life-threatening bleeding episodes, she refused vaginal approaches for diagnosis and treatment, which made the case more challenging. In this case, we managed the patient while preserving her autonomy.

Ultrasound remains the most employed initial modality for investigating abnormal vaginal bleeding. This patient first presented to another facility where she was investigated using a pelvic CT with contrast, which detected the pathology. Few cases in the literature have been diagnosed by CT with contrast as there are more superior imaging modalities. However, CT scans help determine the size, extent, and vascularity of UAVM, along with defining the involvement of adjacent organs such as the intestine or spine [11].

Although transvaginal ultrasound with Doppler is the ideal sonographic approach to diagnose UAVM, transabdominal ultrasound with Doppler was performed in this case, and the findings were consistent with UAVM along with MRI with contrast (Figures 1, 2).

UAVM can be a life-threatening condition causing severe genital bleeding. Our patient lost around 3 L of blood in our facility. Management of profuse vaginal bleeding was tricky as she was a virgin. Additionally, our patient had a previous anaphylactic reaction to the use of tranexamic acid, a commonly used medication to control bleeding. Fortunately, after blood product replacement, her bleeding stopped spontaneously.

It is important to emphasize that UAVM management is highly individualized based on women’s age, desire for future fertility, and severity of bleeding. Given the emerging evidence of the higher success rate of hysteroscopic resection of the malformation and its potential superior outcome in fertility preservation and overall pregnancy outcomes compared to UAE [9], hysteroscopy was offered first, but, ultimately, the patient underwent UAE.

The primary success rate after the first embolization is 79.2% [12]. Our patient remained stable post-UAE. A repeat MRI after two months revealed persistent activity in the left parametrial vessels (Figure 6). This finding could be related to the left ovarian vein varicosity observed on angiography. However, as the patient was complaining of heavy vaginal bleeding on the second day of the menstrual cycle, hysteroscopic resection was offered again while reassuring her about the hymen’s integrity and the repair of any likely damage occurring intraoperatively. Nevertheless, the patient opted for mefenamic acid and remained stable with it.

## Conclusions

UAVM is diagnosed using non-invasive approaches such as transvaginal ultrasound with color Doppler and MRI. However, it is worth noting that UAVM can be detected by transabdominal ultrasound with color Doppler and CT scan if the above-mentioned methods are not feasible. When definitive detection is required, angiography, an invasive procedure, is considered the gold standard. Embolization is the current recommended treatment for women who wish to preserve their fertility. However, there is emerging evidence that hysteroscopic resection is the superior approach. We recommend further trials to compare the efficiency of both approaches for fertility preservation and long-term obstetric complications. Our patient had multiple life-threatening bleeding episodes, but, ultimately, she refused all vaginal methods for management. Hence, we recommend further studies to investigate the level of stigma associated with the integrity of the hymen in the Middle East and identify the public perception of female virginity.
